# Subjective SES is Associated with Children’s Neurophysiological Response to Auditory Oddballs

**DOI:** 10.1093/texcom/tgaa092

**Published:** 2020-12-04

**Authors:** Alexander L Anwyl-Irvine, Edwin S Dalmaijer, Andrew J Quinn, Amy Johnson, Duncan E Astle

**Affiliations:** 1 MRC Cognition and Brain Sciences Unit, University of Cambridge, Cambridge, CB2 7EF, UK; 2 Oxford Centre for Human Brain Activity, Wellcome Centre for Integrative Neuroimaging, Department of Psychiatry, University of Oxford, Oxford, OX3 7JX, UK

**Keywords:** auditory oddball, language development, MEG, phonological processing, socioeconomic status

## Abstract

Language and reading acquisitions are strongly associated with a child’s socioeconomic status (SES). There are a number of potential explanations for this relationship. We explore one potential explanation—a child’s SES is associated with how children discriminate word-like sounds (i.e., phonological processing), a foundational skill for reading acquisition. Magnetoencephalography data from a sample of 71 children (aged 6 years and 11 months–12 years and 3 months), during a passive auditory oddball task containing word and nonword deviants, were used to test “where” (which sensors) and “when” (at what time) any association may occur. We also investigated associations between cognition, education, and this neurophysiological response. We report differences in the neural processing of word and nonword deviant tones at an early N200 component (likely representing early sensory processing) and a later P300 component (likely representing attentional and/or semantic processing). More interestingly we found “parental subjective” SES (the parents rating of their own relative affluence) was convincingly associated with later responses, but there were no significant associations with equivalized income. This suggests that the SES as rated by their parents is associated with underlying phonological detection skills. Furthermore, this correlation likely occurs at a later time point in information processing, associated with semantic and attentional processes. In contrast, household income is not significantly associated with these skills. One possibility is that the subjective assessment of SES is more impactful on neural mechanisms of phonological processing than the less complex and more objective measure of household income.

## Introduction

The ability to decode sound structures within language—sometimes called phonological processing—is a key building block for language acquisition (Wagner and Torgesen 1987; Torgesen et al. 1994; Vihman 1996) and becoming a skilled reader (Wagner et al. 1997). Behavioral measures of language proficiency, reading ability, and phonological processing are all highly related to each other (Nation and Snowling 2004). This broad category of phonological processing can be subdivided into lower-level abilities: phonological awareness, phonological/verbal working memory, and phonological retrieval (Wagner and Torgesen 1987). Here, we focus on the lowest level, “phonological awareness,” which describes the degree to which an individual can perceive, judge, and utilize constituent sounds of language (Hulme et al. 2005). We specifically look at how the processing underlying phonological awareness is associated with socioeconomic status (SES) and behavioral measurements.

### SES is Associated with Phonological Processing, Reading and Language

SES is a factor that captures family or individual income, education, welfare, and cultural capital (McLoyd 1998; Kolenikov and Angeles 2009). In children, SES directly relates to attitudes, cognition, educational outcomes, and mental health (Dalmaijer et al. 2019). However, in terms of effect size, one of the strongest relationships is between SES and language development (Bus et al. 1995; Pungello et al. 2009). Children who grow up in low-income households are more likely to have poorer language skills as an adult (Schoon et al. 2010), show poor reading ability (Buckingham et al. 2014; Noble et al. 2006), and perform poorly on tasks that require phonological awareness (Whitehurst 1997; Noble et al. 2005, 2006, 2007). A recent study by Dolean et al. (2019) drew on a sample of 322 children facing severe poverty in the Roma community and contrasted it with 178 non-Roma children. This study illustrated the core problem: low SES directly negatively impacts reading development, as well as all variables that contribute to it, such as school absence, rapid atomized naming, phonological awareness, letter knowledge, and nonverbal intelligence quotient (IQ).

One influential model proposes that SES also impacts brain development through two parallel paths (Noble et al. 2012; Ursache and Noble 2016). One of these paths posits that SES impacts a child’s language skill through the linguistic environment at home, which in turn leads to structural differences in the brain, specifically in the left inferior frontal and left superior temporal gyri. Another path shows low SES increasing stress, which influences multiple brain areas, and in turn degrades social–emotional processing, memory, and self-regulation. In line with this model, research has shown that SES does indeed moderate the relationship between task-measured phonological performance and left fusiform gyrus activation. Noble et al. (2006) selected children matched in phonological skill, but from a range of SES backgrounds and had them perform a pseudo-word reading task during functional magnetic resonance imaging (MRI). Lower SES children’s brain activity appeared to moderate task performance, whereas higher SES children showed an attenuated relationship. This was apparent in the left fusiform and perisylvian regions. These brain regions were selected a priori for the regression analysis, so there may have been a wider pattern across brain regions—and the relationship in Noble’s model may extend beyond the fusiform gyri.

In more recent work, Younger et al. (2019) reveal that greater maternal education (ME) (an element of SES) is associated with different patterns of brain lateralization in 5-year olds. Increased ME was related to higher brain lateralization toward the left inferior frontal gyrus. Furthermore, this interacted with phonological awareness performance, such that performance was related to a leftward bias in the superior temporal gyrus in low ME children, but with a rightwards bias in high ME children. These results suggest that an SES factor (ME), impacts actual neural recruitment during language processing—supporting the concept of an SES-moderated language developmental path in the brain.

Based on this prior work, it is therefore uncontroversial that phonological skill, and related processes, is influenced by a child’s SES (Hoff-Ginsberg 1998; Pungello et al. 2009). As alluded to above, there are many possible mechanisms by which a child’s environment could influence this set of processes. One possibility is that SES is associated with the ability to discriminate word-like sounds. We test this in the current study, by measuring the neurophysiological response to passively perceived sound structures using magnetoencephalography (MEG). Specifically, we looked at the response to irregular word sound structures against frequent nonword sounds—representing sensitivity to the words. We investigated at which “time points” and “locations” in the information processing stream this neurophysiological process is influenced by a child’s SES. Our whole brain/sensor analysis approach allows us to build on the a priori area selection findings from work such as Noble et al. (2006) and Younger et al (2019), by potentially revealing new areas that relate to SES. Furthermore, as SES captures such a variety of factors, we also split our measures into two aspects: one reflecting the absolute financial means available to the child’s family, and another using a subjective rating of the families means. Previous work has shown that subjective and objective measures of SES make independent contributions to children’s executive functions, stress, and cortisol (Ursache et al. 2015).

### Auditory Oddballs and Phonological Processing

It is helpful to characterize the utility of the oddball paradigm for this type of research. An oddball tasks consist of sequences of repetitions of a “standard” stimulus, interspersed with infrequent deviant stimuli. Comparing the neural response of the subject’s brain to frequent and infrequent stimuli provides a measure of whether and when those stimuli are detected as different by the brain (Dehaene-Lambertz and Gliga 2004), independent of whether they were attended or consciously perceived (Schröger 1997). The observed difference in response between standard and deviant stimuli (“mismatch signal”) relies on networks of neurons adapting to a repetition of input by suppressing their activation and then releasing from this adaptation when a change is detected (Naccache and Dehaene 2001). In MEG and electroencephalography, this signal leads to a negative peak at roughly 200 ms, termed mismatch negativity (MMN), and later components such as the P300, which is associated with further semantic (Meador et al. 1987) and attentional processing (Bennington and Polich 1999).

Oddball experiments have been deployed by researchers to investigate the underlying mechanisms of phonological awareness in both children (Cheour et al. 2001; Korpilahti et al. 2001; Linnavalli et al. 2017) and adults (Näätänen 1990). Additionally, a large literature investigates specific conditions, for example, autism (Oram Cardy et al. 2005), dyslexia (Wehner et al. 2007), specific language impairment (Shafer et al. 2005), or community samples, such as poor readers (Bernal et al. 2000). In contrast, there is little research on the impact of SES on oddball-evoked responses, especially in typically developing children. One study utilized a visual oddball (i.e., a novel picture in a stream of standard shapes) in a group design, with 26 subjects aged 7–12 years, split between low-SES and high-SES groups. It found attenuated early mismatch responses, but no SES-related P300 differences (Kishiyama et al. 2009). The inclusion of only 13 children in each group of this study potentially obscures any subtler relationships between the mismatch effect and SES. In fact, developmental auditory oddball studies often have smaller sample sizes and/or group designs that potentially limit sensitivity, for example, Korpilahti et al. (2001) *N* = 10, Lovio et al. (2009)  *N* = 17, Cao et al. (2008)  *N* = 12 per group, Bakos et al. (2016)  *N* = 14 and *N* = 15 in each group, and Orinstein and Stevens (2014)  *N* = 18 and *N* = 20 in each group.

### The Current Study

In the current study, we tested whether the neurophysiological mechanisms, by which simple word-like sounds are distinguished, vary according to a child’s SES. We used a passive oddball task to test this. Children sat in the MEG scanner while watching cartoons. During their viewing, they listened to trains of sounds containing carefully matched oddball words and nonwords alongside fillers. The children also took part in a structural MRI scan, allowing us to try and localize the MEG activity to a brain model created from their scan.

We recruited children and their families to take part in a MEG and MRI scan, from a wide variety of household incomes (range £5700–£66 000 annual household income). The age range was from just under 7 years old to just over 13 years—a wider range than previous studies such as Kishiyama et al. (2009). This may allow us to capture more developmental changes. Additionally, it expands on the phonological electrophysiology literature that focuses on earlier ages (<5 years) when these systems are just developing.

We used a general linear model (GLM) that included behavioral and demographic variables to predict evoked neural activity in three dimensions (time and 2D space) during the phonological oddball task. This tests how variance across the whole group predicts the underlying neural activity, as opposed to the limited single contrasts in group designs. This GLM allowed us to take a data-driven approach, asking whether a child’s SES is associated with their neurophysiological response to carefully matched words, and crucially, if so, “when” this influence occurs. One possibility is that SES will covary with the earliest neurophysiological response to an oddball (Korpilahti et al. 2001). Alternatively, it may covary with a later processing stage more likely to reflect order, semantics, or attentional processing (Meador et al. 1987; Bennington and Polich 1999; Hill et al. 2004). Our GLM will enable us to detect either, or both of these effects, if they exist.

SES was characterized using equivalized household income as an objective measure, and parent’s self-reported SES as a subjective measure. Parental education level is another potential metric used within the literature, but it has relatively few levels (high school, university degree, higher education) by comparison with the other SES metrics. We also collected behavioral data from these children: measures of educational attainment in reading and maths, and cognitive measures of working memory, verbal skills, and general IQ. These were incorporated within the GLM to test whether these individual differences were also associated with the phonological processing of word oddballs, independent of SES.

## Materials and Methods

### Participants

A total of 82 participants took part in the study, conducted at the MRC Cognition and Brain Sciences Unit. Due to technical problems with the scanner (4 children), attrition between sessions (2 children), and children opting out of either MRI or MEG (5 children) scan only 71 full datasets remain. There were two visits for each child; on the first, behavioral measures were collected, and then a MEG scan took place. On the second (which was optional) the participants had a structural MRI. There was no more than a month between visits.

The mean age of the children was 9 years and 11 months (range: 6 years and 11.6 months–12 years and 9.3 months), 44 of the children were boys. We computed the average net household equivalized income, which is income after tax deductions and benefit additions, weighted by number of children and adults using Organisation for Economic Co-operation and Development (OECD) equivalence scale (Anyaegbu 2010). This was £24 313 on average, with a standard deviation of £12 261, ranging from £5747 to £66 666. Our sample was thus socioeconomically diverse, but of lower means than the UK median at time of testing (£31 876), 2017/18. In fact, 26.8% (22 children) were living under the UK poverty line–classified as 60% of the median income or less (“households below average income” 2018). All our families live in the Cambridges and East Anglia area, where the cost of living is high by UK standards, so it is likely that this statistic underestimates the proportion living below the poverty line. We did not record the ethnicity of our participants; however, the vast majority of Cambridge (82.51%) and east of UK (85.1%) (Office for National Statistics 2018) are White, and we were unlikely to have recruited enough of other groups for meaningful statistical inference.

A questionnaire was given to parents to ascertain subjective SES, obtained by having caregivers place a cross on a ladder of 10 rungs, with the top representing those who were better off in the United Kingdom, and the bottom representing those the worse off. This is a frequently used measure of subjective SES (e.g., Ostrove et al. 2000; Singh-Manoux et al. 2005).

### Procedure

Volunteers and their families took part in all research sessions at the Medical Research Council Cognition and Brain Sciences Unit, University of Cambridge. Parents provided written informed consent, and children provided verbal assent. The study was approved by the Psychology Research Ethics Committee at the University of Cambridge (Reference: 2015.11).

### Behavioral Measures

Children and their families visited the Unit for a battery of educational attainment and cognitive assessments. These included mathematics and reading fluency scales from the Woodcock–Johnson III Form B tests of achievement (Woodcock et al. 2001), the matrix reasoning and vocabulary subtests of the Wechsler abbreviated scale of intelligence (WASI-II) (McCrimmon and Smith 2013), the automated working memory assessment (AWMA) (Alloway et al. 2008), and the phonological assessment battery (PhAB) (Gallagher and Frederickson 1995).

### Phonological Oddball

#### MEG Scan

During the first visit, neuroimaging data were acquired on a high-density VectorView MEG System (Elekta Neuromag) with 102 magnetometers and 102 orthogonal pairs of planar gradiometers (306 sensors in total). Head position indicator (HPI) coils were attached to the child’s head (one on each mastoid bone, two on the child’s forehead, and one on the top of their head). A 3D digitizer was used to record the positions of each HPI coil, and a number of scalp points (50+) in order to assist in coregistration of MRI scans. To capture eye-movements and blinks, vertical and horizontal electrooculogram (EOG) were measured with a pair of electrodes to the side of each child’s eyes, and another pair placed above and below the left eye. To record heart rate, an electrocardiogram (ECG) was taken with electrodes attached to each wrist. Audio was presented to the participants using in-ear earpieces attached to a long plastic tube that went outside the MEG’s shielded room, where they were attached to the speaker and amplifier. This minimized the impact of any electrical signal from audio amplification and production.

#### MRI Scan

During a separate visit, participants took part in an MRI scan, which yielded T1-weighted images from a Siemens 3T Tim Trio system. For these images, a magnetization prepared rapid acquisition gradient echo sequence with 1 mm isometric image resolution 2.98 ms echo time and 2250 ms was used.

#### Task

Three auditory stimuli were used: a novel pseudo-word frequent (“boak”), a known word oddball (“boat”), and a novel pseudo-word oddball (“boap”). The ratio between these stimuli was 6:1:1, that is, one of each oddball for every six frequent stimuli. The task started with a train of 10 standard stimuli, so that participants could habituate to the frequent nonword. There were 1200 trials in total (900 nonword standard, 150 word oddball, 150 nonword oddball). In a pseudo-random manner, there were either 2, 3, 4, or 5 standard nonword stimuli between deviants. The interstimulus interval was 800 ms from the offset of one stimulus to the onset of the next.

The stimuli themselves were taken from (Hawkins et al. 2014). All words had identical first consonant-vowel, /bo℧/(“boa”), which was spliced from natural spoken word taken from speaking the word /bo℧t/ (“boat”). For each stimuli this sound was then cross-spliced with a voiceless-top consonant, that was either: /k/ to make standard nonword /bo℧k/ (“boak”), /t/ to make oddball word /bo℧t/ (“boat”), or **/**p/ to make oddball nonword /bo℧p/ (“boap”). The first consonant-vowel was acoustically and coarticulatory identical until the final stop vowel, and peak sound energy was equated across all stimuli. This meant that the ability to perceive the sounds as different only happened at the last phoneme, which should target as exclusively as possible the systems underlying phonological awareness.

During the oddball task, all children watched a cartoon (Tom and Jerry: The Classic Selection Volume 1) (Takeda and Kimura 2014), without any audio. This particular cartoon had the benefit of not having any moving mouths for speech—so visual speech cues would not confound or convolute signal from the auditory cortex (Sams et al. 1991). It also kept the children relatively entertained during the scanning session.

### Analysis

Data were analyzed primarily with the MNE-Python toolbox v0.19 (Gramfort et al. 2013) on CentOS Linux.

### Preprocessing

Raw data underwent signal source separation, temporal extension, and movement compensation using Maxfilter 2.2. These data were loaded into MNE-Python, and then high-pass filtered at 1 Hz and low-pass filtered at 50 Hz. In order to remove noise associated with heart beats and blinks, a two-stage independent component analysis (ICA) denoising procedure was used. An ICA was done using “fastica” with 25 components specified. Stage 1 involved automatic rejection of components that correlated with ECG or EOG electrodes more than 0.3. Stage 2 involved manual checking of excluded component topography and selection of components to exclude for participants with insufficient ECG or EOG electrode signal. Data for each child were visually checked before and after to ensure the components were not present still.

Raw data were then epoched between 200 ms before and 1000 ms after the presentation of auditory stimuli. As participant data were split up into two runs, these were processed separately until epoching, where epochs were concatenated and treated as one after this.

### Source Localization

FreeSurfer (Fischl 2012) was used to construct whole brain surface from MRI scans, using the recon-all command. A single layer boundary element model (BEM) of the inner skull was constructed using the MNE watershed method. A source space was made using the cortical surface from the FreeSurfer output. Our inverse model consisted of this one-layer BEM, and the method used to invert the evoked signals was the MNE toolbox’s implementation of dynamic statistical parametrical maps (DSPM), with empirical whitening done using a noise-covariance matrix taken from the baseline period, which we found to produce the most consistent results. Participants who lacked an MRI or moved too much during the MRI scan had models created using FreeSurfer’s FSAVERAGE model.

### Behavioral Statistical Analysis

We had a large number (12) of likely highly corelated behavioral measures. This multicollinearity makes using these predictors in our later GLM inappropriate. Consequently, these were reduced to separate components using principal component analysis with orthogonalization through varimax rotation. Behavioral variables (Woodcock–Johnson III subtests, AWMA, and WASI-II) were reduced to 3 factors, which we labeled working memory and executive, classic IQ, and verbal short term memory (STM) and working memory (WM)—these were chosen as plausible factors based on previous work (Alloway et al. 2005) and explained 45.5% of total variance. Education (the Woodcock–Johnson III measures) was subject to a separate factor reduction. Parallel analysis revealed that in the best solution Woodcock-Johnson (WJ) reading and mathematics was a single factor solution, explaining 47.8% of variance in those scores. The factor weightings can be seen in Table 1. Even though the WJ were used to derive a single factor, we show correlations between all the components/factors and the scores. You can see that the WJ scores correlated with some of the other three factors; however, they did not contribute to those factor scores.

**Table 1:** The factor weightings for each of the component scores extracted

	Working memory and executive	Classic IQ	Verbal STM and WM	Attainment
AWMA digit recall	−0.05	0.23	0.71	0.24
AWMA dot matrix	0.62	0.02	0.00	0.00
AWMA Mr X	0.60	0.36	−0.03	0.02
AWMA backward digit	0.17	0.28	0.50	0.12
WASI vocabulary	0.02	0.74	0.26	0.36
WASI matrix reasoning	0.42	0.59	0.19	0.28
WJ reading	0.10	0.48	0.36	0.50
WJ mathematics	0.24	0.33	0.34	0.45

Note: This is shown as each component variables Pearson correlation with the factors. The WJ subtests were used to derive the “Attainment” component, but excluded from the other components—correlations across all components and scores are still included for completeness.

We did not include scores from the PhAB alliteration measure; this task was too easy for children of this age, without phonological awareness difficulties. Fifty-one out of 71 (70.4%) of the children answered all items correctly, so showed little variance. We used age standardized (WASI *t* scores, AWMA, and Woodcock–Johnson standard scores) scores in all or our analyses, with age in years then added as a covariate in the later GLM, such that age would be independent against all measures.

### MEG Statistical Analysis

#### Comparison of Word and Nonword Contrasts

In order to investigate whether there was a significant difference between the word and nonword MMN, a nonparametric, cluster corrected, two-tailed repeated measures permutation *t*-test was calculated using the difference field between the two. A connectivity matrix was computed over time and space, and a cluster forming threshold of *t* = 4 was also used to calculate the clusters. This was much higher than the critical *t* of 2 calculated from an effect size (0.28) reported in a meta-analysis of oddball tasks in children (Cheng et al. 2016) with an error probability of 0.05 and a sample size of 71. The threshold is statistically arbitrary, since it is repeated in each permutation (Friston et al. 1994), but having a narrower definition of clusters makes them far easier to interpret in terms of their spatial extent. The permutation test produces null-distributions of cluster *t* statistics based on shuffling data, which is then compared with the actual observed cluster *t* values. This is more computationally demanding than false descovery rate (FDR) methods; however, it is also more conservative and has the benefit of directly controlling the family-wise error rate, rather than the FDR statistic (Nichols and Hayasaka 2003; Lage-Castellanos et al. 2010). We used a Monte Carlo *P* value of 0.05 to identify significant clusters over 5000 permutations—in other words, clusters identified were in the 95th percentile or higher.

#### General Linear Model

A mass multivariate GLM was constructed to analyze the three dimensional (2D sensor-space x time) average evoked responses for each individual in relation to the behavioral factor scores (in Table 1), along with age (in days), equivalized income and subjective SES. This allows us to test how individual’s spatio-temporal responses predict their cognitive, attainment, and demographic attributes. For the neurophysiological data, we used only the word contrast (i.e., word vs. nonword fillers), as this represents the sensitivity to word phonological forms, rather than the nonword contrast, which is concerned only with sensitivity to sounds unrelated to real words.

A design matrix was constructed with each row containing a continuous regressor of value 1, representing a single participant’s word contrast (102 magnetometers in 2D space × 1200 ms time samples), and a single value regressor for each of working memory and executive factor, classic IQ factor, verbal STM and WM factor, attainment factor, age in years, equivalized income, and subjective SES. All regressors were *z*-transformed (so they were normalized and centered around zero). The final design matrix was thus 71 × 8.

In order to find our best estimates of the model’s betas, we used ordinary least squares to minimize the models error terms. This resulted in beta weights for each predictor at each point in time and space. These beta values (and statistics inferred from them) represent the relationship between regressor and evoked response for each time point. Larger values reflect a stronger relationship at that spatio-temporal measurement.

We then took a cluster permutation approach to establish inference from our model. The *t* values were calculated for each beta value, and spatio-temporal clusters (2-tailed) were extracted from this (as in the previous analysis), and the mean *t* value taken. We found that the cluster forming threshold of 4 yielded large numbers of small clusters, so reduced the value to create larger more interpretable clusters before permuting. This was a statistically arbitrary cluster forming threshold of 2.8. As before, 2.8 was higher than the critical *t* value for an expected effect size of 0.28, based on an oddball meta-analysis of children (Cheng et al. 2016), with a sample size of 71 and an alpha of 0.05.

We then permuted each of the 9 regressors in the model 5000 times (45 000 total permutations), where the rows of that regressor were randomly shuffled while holding covariates constant (so they no longer matched the participant’s data), spatio-temporal clusters were recalculated, and the average *t* value taken. This gave us a Monte Carlo distribution for each regressor that was centered at zero, which was compared with the original clusters. Any original (unshuffled) cluster with a value in the 95th percentile of the Monte Carlo distribution was kept as a significant cluster.

## Results

### Group Level MMN Evoked Response

**Figure 1:**
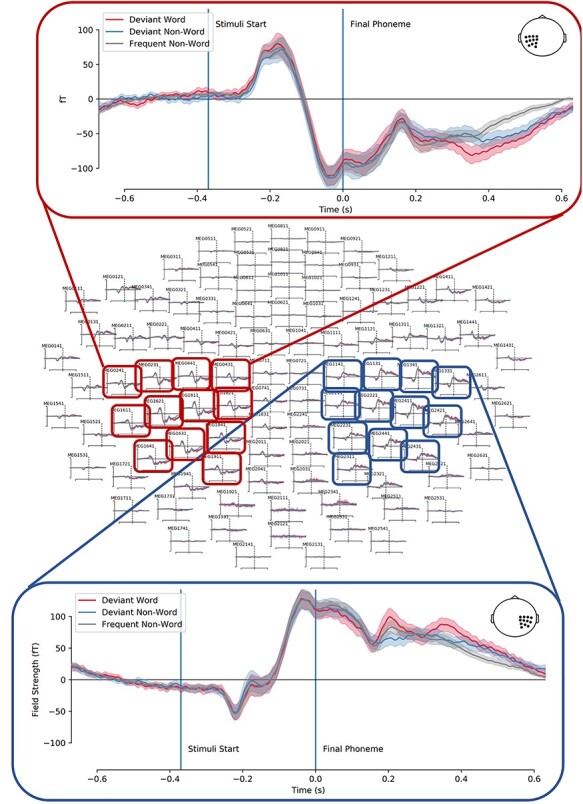
Illustrative topography and time course of the evoked responses for the frequent nonword and the word/nonword deviants. A subsection of left and right sensors was selected and averaged to produce the time courses above and below the helmet illustration.

@Evoked responses for the nonword frequents, nonword deviants, and word deviants can be seen for all magnetometers in Figure 1**.** This figure is purely illustrative; it shows the evoked signal for each trial type before subtractions on a handful of representative electrodes. For reference, we identify the beginning of the sound, and the differentiation point (the final phoneme) on all points. Based on the topography of these responses, we selected right and left parietal sensors that showed the clearest apparent auditory evoked topography (the mean of these sensors is also illustrated in Figure 1).

There is a clear auditory evoked component at around 150 ms after the onset of the sound, the direction in power compared with baseline is positive on the right sensors and negative on the left sensors. There appears to be a difference in the evoked responses to the oddballs and the frequent stimuli that begin appearing around 200 ms after the onset of the final phoneme, with more pronounced differences by 400 ms. In order to test this statistically, we compared deviant minus frequent subtractions for the words and nonwords. All sensors and time points were entered into a cluster-permuted *t*-test and detailed in the methods section.

**Figure 2:**
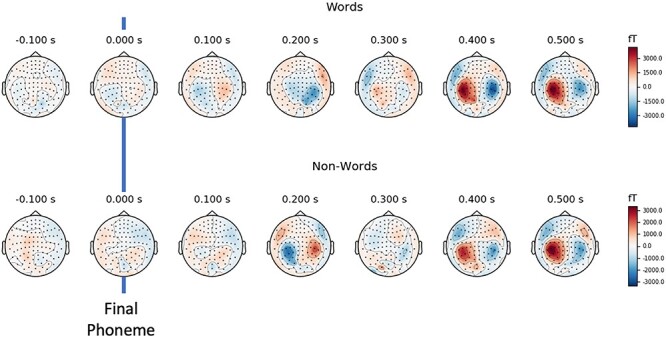
Field strength topography of evoked contrasts for words and nonwords. Final phoneme is marked with a blue line.

There were clear differences between the two different mismatch contrasts: words (i.e., word deviants, relative to nonword frequents) and nonword (i.e., nonword deviants relative to nonword frequents). In sensor space, the evoked topography for these word and nonword contrasts is plotted in Figure 2. There is a clear pattern of left and right parietal activation at the 400 and 500 ms bins, where results are (qualitatively) similar between contrasts. At the 200 ms bin, we see a unilateral decrease for the word contrast in the right parietal area, and this pattern is reversed in the nonword contrast.

**Figure 3:**
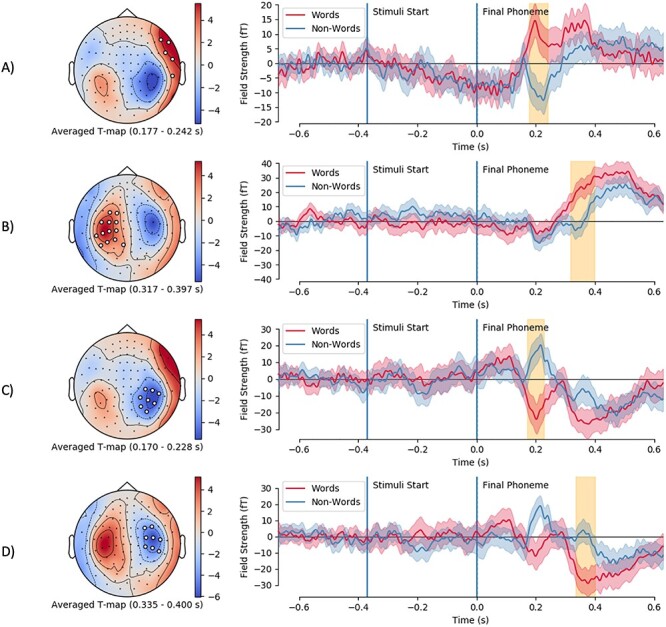
Evoked field cluster topography and time courses for word and nonword mismatch subtractions. Mean *T* statistic maps are shown projected onto MEG helmet, significant sensors are marked in white. Time course showing mean (line) and bootstrapped 95% confidence intervals (shaded area) field strength for each evoked contrast, stimuli start and final phoneme onset marked in blue, and cluster onset/offset shaded in yellow. (*A*) shows MMN for nonword deviants, and (*C*) shows MMN for word deviants; (*B*) and (*D*) show later difference in response.

The binned topography is a very coarse metric. Greater granularity is provided by looking at the spatio-temporal clusters from the cluster-permuted *t*-test. Four spatio-temporal (i.e., sensor time point) clusters survived permutation testing; these are illustrated in Figure 3: *A* shows a right-temporal topology with a higher response to word deviants versus nonword deviants at 177–243 ms, *B* shows a left-parietal response in the same direction (word deviants higher than nonword deviants) higher later at 317–398 ms, *C* shows a right-temporal topology with nonword deviants responding higher in the same temporal pattern as 2.a at 170–229 ms, and *D* shows a very similar topology and relationship to Figure 3C but later on at 335–401 ms. As mentioned above, these locations and times are a coarse indication of the “true” effect as we have not permuted these dimensions. More detailed statistics on the clusters are available in Table 2**.**

**Table 2:** Statistics for the evoked cluster

	A	B	C	D
Mean *t* value	0.1480	0.0776	0.0373	−0.1101
Monte Carlo *P*	0.0004	0.0002	0.0002	0.0002
Number of sensors	5	13	8	8
Epoch start time (ms)	177	317	170	335
Temporal extent (ms)	66	81	59	66

Note: Mean *T* value is calculated by averaging the observed *T* output from the test statistic at each time point and each sensor in the cluster.

**Figure 4:**
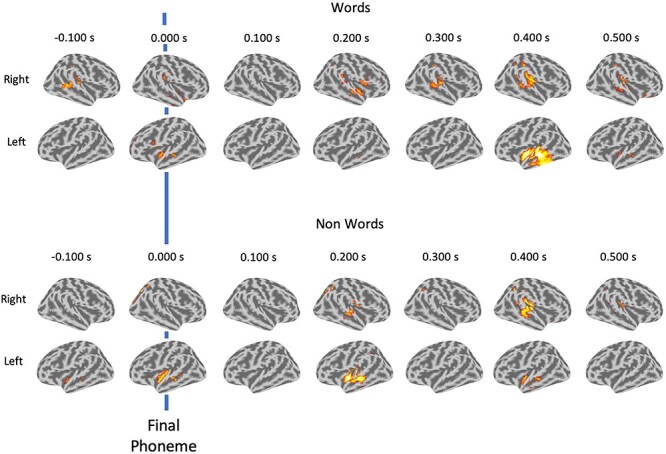
Average source-localized evoked contrasts for words and nonwords. Final phoneme is marked with a blue line. DSPM used to invert sensor-level data. For visualization the estimates are binned into 100 ms segments, so each image is a mean average across a bin.

Although not critical for our core research questions, we were interested in where these responses originated from. Quality source-reconstruction was possible for 47 of our participants—this was not high enough to go through with source analysis. However, we are able to show the average topology for these participants. Figure 4 illustrates the likely origins of the mismatch response. This replicated not only the sensor-level data, but also shows the word contrast more prominently localized to the left anterior temporal lobe at approximately 400 ms compared with the nonword contrast.

### Group Level Behavioral GLM

The attainment factor, age in years, and subjective SES regressors all yielded clusters that were robust to our permutation testing (Table 3). The predictors working memory and executive factor, classic IQ factor, verbal STM and WM factor, and equivalized income did not survive this testing, and we found no evidence for a relationship between these variables and the MMN response.

**Table 3:** Summary of statistics for GLM clusters surviving permutation testing

	Attainment	Age in years	Subjective SES #1	Subjective SES #2
Mean *beta*	2.2929E-14	−1.5227E-14	−1.0352E-14	−1.9247E-14
Mean *t* value	3.0505	−3.3638	−3.1600	−3.1044
Monte Carlo *P*	0.0222	0.0462	0.0460	0.0474
Number of sensors	15	12	20	20
Epoch start time (ms)	463	516	3	340
Temporal extent (ms)	185	106	72	77

Note: *T* values and Beta values are the average from each cluster over sensors and time points. Beta values are in the scale of magnetometers field strength.

**Figure 5:**
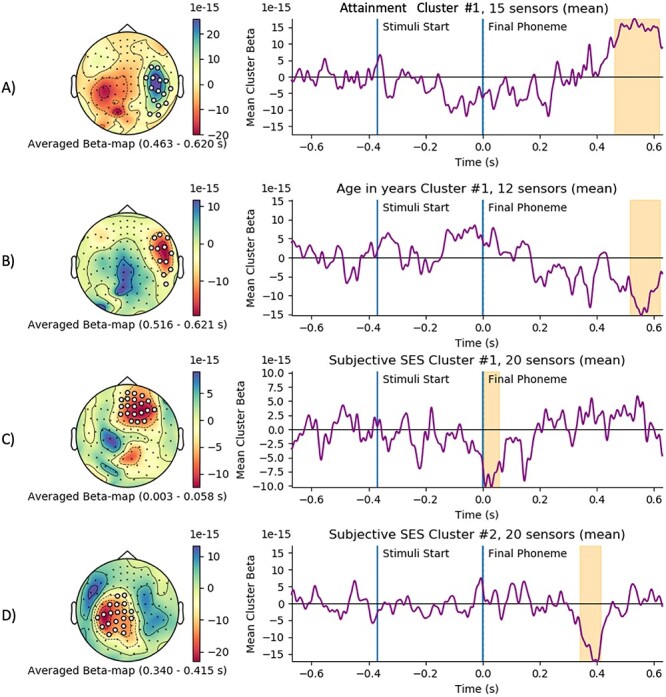
General linear model clusters for attainment (A), age in years (B), and Subjective SES (C & D). Topography of beta-weights with cluster sensors plotted shown on the left. Time course of beta weights, with stimuli and final phoneme marked in blue, and cluster temporal extent shaded in yellow. It should be noted that spatial and temporal cluster extent are not cluster permuted, just the statistic, so this should be interpreted as an estimate of these dimensions. Beta values are in the scale of magnetometers field strength.

The topography of three of the clusters (Fig. 5*A*,*B,D*) showed clear overlap with the evoked response shown in the results above, whereas the third cluster (Fig. 5*C*) did not overlap with this temporally or spatially. The education cluster (Fig. 5*A*) had a right-parietal topography, started around 460 ms after the differentiation point, and predicted an increased response to word oddballs against nonword frequents. The age cluster (Fig. 5*B*) showed a right-temporal topography, started around 500 ms, and predicted a decreased response to word oddballs versus nonword frequents. The first subjective SES cluster (Fig. 5*C*) had a frontocentral topography, an unexpected time course that started at the differentiation point (with an onset just after differentiation) and predicted a more negative response to oddball words relative to frequent nonwords. The second subjective SES cluster showed a more plausible time course and topology, with a left-parietal topology starting around 350 ms after the differentiation point, and predicted a more negative response to oddball words versus frequent nonwords. We report the temporal and spatial elements of these clusters roughly, as these dimensions of the clusters are estimates (Sassenhagen and Draschkow 2019).

## Discussion

We used an auditory oddball paradigm to explore the relationship between children’s sensitivity to phonological deviations and their SES. Measures of cognition and educational attainment were also included in the model. Children showed a robust and differential response to the final phoneme of word deviants versus nonword deviants. The significant clusters of difference were at ~ 200 ms, with two clusters showing opposing responses to words versus nonwords—both on the right hemisphere, and then at ~ 350 ms showing the same polar differences, but with a contralateral topography. Importantly, a child’s subjective SES is associated with their neurophysiological response to deviant words, and one cluster showed overlap with a later P300 response. Attainment and age also show statistically significant associations with the evoked response to word deviants, and these clusters occurred later, also consistent with a late P300 component. There was no evidence that these factors are associated with the earlier N200 response, and there was no evidence for cognitive measures or household income to be associated with the evoked response.

### Differences in Word and Nonword Contrasts

We report components that show a difference between the word and nonword contrasts, a N200 and P300 component. The N200, or MMN, component implies that there is an early sensory detection between the processing of unexpected word and nonword phonemes—this was expected and replicates previous observations (Korpilahti et al. 2001; Maurer et al. 2003; Junge et al. 2012). The P300 component is commonly associated with conscious processing and attentional orienting (Sommer and Matt 1990; Bennington and Polich 1999; Polich 2007). Despite the explicit instructions to ignore the stimuli and focus on the simultaneous cartoon playback, it is likely the irregular stimuli led to an involuntary orienting of attention (Lyytinen et al. 1992). The differences between word and nonword contrasts are therefore likely to reflect some degree of differing involuntary attentional shifting, or at least an increased demand on attention (Bennington and Polich 1999), and the neural processing associated with this. Another strong possibility is that this component is associated with semantic processing (Meador et al. 1987) and phonological categorization (Hill et al. 2004), perhaps indicating that this later difference could also reflect differing processing of semantics and categorization—which is likely given that our contrast of interest is between words and nonwords.

### Subjective SES is Associated with the Oddball Response

A child’s oddball response was not significantly associated with equivalized family income, but it was significantly associated with parental rating of subjective SES. We conclude from this that the economic situation per se is not the ingredient that drives SES-phonology associations, but instead that it is the wider environmental impact of SES, which the parent is uniquely placed to assess. Greater relative deprivation, which cannot be completely captured by standard measures like income, likely negatively impacts the development of phonological processes—the subjective SES effect may well reflect this. An alternative explanation is that lower subjective SES is associated with poorer parental mental health, which in turn leads to less support for language development and therefore phonological processing. Supporting this second explanation, lower subjective SES in adults is indeed associated with poorer mental health (Scott et al. 2014; Odgers and Adler 2018), and poor parental mental health is negatively associated with early (1–2 years) language development (Lung et al. 2009; Paulson et al. 2009). We did not measure parental mental health, but this may be a potential mediating factor and would provide a future direction for research.

Irrespective of the explanation, the results speak to the complex nature of SES, which is often characterized as purely with income or occupation (Rubin et al. 2014). As income was included as a predictor in the GLM, it is likely that the subjective SES clusters represent variance independent of income. Indeed, this observation partly parallels research into children’s executive functions where objective SES and subjective SES were shown to make independent contributions (Ursache et al. 2015). Across the literature, the way we conceptualize SES seems to be crucial. When ME is used to group children, differences in selective attention (Stevens et al. 2009) and auditory refractory periods (Stevens et al. 2015) are observed. In contrast, grouping by income alone does not always produce significant differences (Garcia-Sierra et al. 2011). Taken in concert with our results, this could also support one path of the theoretical model put forward by Noble et al. (2012) and Ursache and Noble (2016)—that language and phonological development are moderated by “some elements” of SES and impact later out comes in children. In our case, it seems to be the subjective experience of SES, rather than income per se.

We found two SES-predictive clusters that survived permutation testing. One frontal-central cluster that starts very early (almost at the differentiation point), and a second more left-dorsal cluster that has an onset consistent with a P300 component. We are dubious about the first of these. Only the cluster statistics are permuted, not their spatial or temporal extent—meaning we cannot make statistical inferences about the precise time and space (Sassenhagen and Draschkow 2019). The shape and location of clusters are liable to display spreading. This limitation can explain the first SES cluster (Fig. 5*C*), which appears implausibly early. It is possible that the true effect has occurred later, and by chance, the original cluster had been formed in its current location. If this is the case, this may indicate an association with earlier sensory processing in reaction to the word oddballs, perhaps in relation to observations of auditory ventral stream processing reported in oddball tasks (Kim 2014). However, due to its dubious time course, this is unclear.

The second cluster (Fig. 5*D*) is more easily interpretable as the topology and timescale overlap highly with the left late P300 component shown in Figure 3*B*. A reasonable interpretation is that subjective SES associated with the process of attentional orienting and/or semantic processing referred to above. In contrast with this finding, altered development of language systems—either through low SES or in children with neurodevelopmental conditions—has often been ascribed to early sensory differences. For instance, Stevens et al. (2009) reported that low SES children showed reduced evoked activity from selective attention to spoken stories at around 100 ms post cue. Our results do not replicate this type of early sensory finding. However, our subjects are relatively old, and it could be that we would see this kind of early effect in younger children, but that its timing is developmentally specific. The later effects that we observe are, however, consistent with some findings in the dyslexia literature. Dyslexia prevalence increases with lower SES, and dyslexic children and adults show altered P3 responses and long latency evoked response potential (ERP) during reading and rhyming tasks (see Taylor and Baldeweg 2002). However, there may be many factors that explain this relationship between the later neurophysiological response and subjective SES, including important mediating factors that we did not measure. Identifying these factors could provide necessary information as to the mechanistic origins of this association.

### Educational Attainment but not Cognition is Associated with Oddball Responses

From our behavioral measures, only the attainment factor (weighting primarily on the mathematics and reading WJ scores) was associated with MEG signal, rather than any of the factors that encompassed STM/working memory and IQ assessments. We think this is likely because we do not have good phonological awareness measures in our cognitive battery. We included the alliteration measure from the PhAB); however, we discovered this contained many ceiling effects. These ceiling effects have also been reported in previous studies (Wheldall and Pogorzelski 2003). This is likely because of the age of our participants, as the PhAB measures are typically sensitive to individual differences earlier in development (Cronin and Carver 1998; Anthony et al. 2007; Furnes and Samuelsson 2011). One possibility is that the educational attainment measures are strongly associated because they in part reflect the longer-term outcome of these earlier differences. This is somewhat compatible with research showing that younger children’s phonological abilities predicted their numerical competency and literacy (Krajewski and Schneider 2009).

### Study Limitations

There are several limitations in our study. Firstly, as outlined above, cluster permutation testing permutes the test-statistic, but not the spatiotemporal aspects of the clusters themselves—thus the time course and sensors in the cluster should be used as a general indication rather than a formal test of these attributes. A second limitation is the age of our participants. They are mid-primary school age to early secondary school, and arguably there could be strong relationships between phonological sensitivity and our factors earlier in development.

Lastly, our analysis approach—using a GLM—identifies how evoked brain data are associated with regressors. Although we select a wide range of regressors, our reported relationships could be explained by any number of unseen covariates, such as parental mental health as we mentioned earlier. However, this is broadly true for any model on these types of data—the regressors included are not exhaustive. Nonetheless, we believe our results are still important. The next step is to understand more precisely which elements of subjective SES may be the active ingredients in shaping the relationship with phonological detection skills.

## Conclusion

Children have a differential neurophysiological response to word versus. nonword deviants in a phonological oddball task. These differences arise at both the N200 and P300 components, likely reflecting differences in early perceptual sensitivity, and later semantic processing and attentional orienting systems. The P300 components of the word condition were predicted by measures of age, attainment, and the families’ ratings of their SES, but not by cognitive measures or household income. This shows that complex demographic measures like SES are predictive of the underlying mechanisms involved in phonological processing, and specifically effecting (for the most part) the later stages associated with semantic processing and involuntary attentional orienting.

## Notes

We thank Matt Davis and Kate Baker for their feedback and discussions regarding the article. *Conflict of interest*: None declared.

## References

[ref1] Alloway TP, Gathercole SE, Kirkwood H, Elliott J. 2008. Evaluating the validity of the automated working memory assessment. Educ Psychol. 28(7):725–734. doi: 10.1080/01443410802243828.

[ref2] Alloway TP, Gathercole SE, Adams A-M, Willis C, Eaglen R, Lamont E. 2005. Working memory and phonological awareness as predictors of progress towards early learning goals at school entry. Br J Dev Psychol. 23(3):417–426. doi: 10.1348/026151005X26804.

[ref3] Anthony JL, Williams JM, McDonald R, Francis DJ. 2007. Phonological processing and emergent literacy in younger and older preschool children. Ann Dyslexia. 57(2):113. doi: 10.1007/s11881-007-0008-8. 18058023

[ref4] Anyaegbu G . 2010. Using the OECD equivalence scale in taxes and benefits analysis. Econ Labour Market Rev. 4(1):49–54. doi: 10.1057/elmr.2010.9.

[ref5] Bakos S, Töllner T, Trinkl M, Landes I, Bartling J, Grossheinrich N, Schulte-Körne G, Greimel E. 2016. Neurophysiological mechanisms of auditory information processing in adolescence: a study on sex differences. Dev Neuropsychol. 41(3):201–214. doi: 10.1080/87565641.2016.1194840. 27379950

[ref6] Bennington JY, Polich J. 1999. Comparison of P300 from passive and active tasks for auditory and visual stimuli. Int J Psychophysiol. 34(2):171–177. doi: 10.1016/S0167-8760(99)00070-7. 10576401

[ref7] Bernal J, Harmony T, Rodríguez M, Reyes A, Yáñe G, Fernández T, Galán L, Silva J, Fernández- Bouzas A, Rodríguez H, et al. 2000. Auditory event-related potentials in poor readers. Int J Psychophysiol. 36(1):11–23. doi: 10.1016/S0167-8760(99)00092-6. 10700619

[ref8] Buckingham J, Beaman R, Wheldall K. 2014. Why poor children are more likely to become poor readers: the early years. Educ Rev. 66(4):428–446. doi: 10.1080/00131911.2013.795129.

[ref9] Bus AG, van IJzendoorn MH, Pellegrini AD. 1995. Joint book reading makes for success in learning to read: a meta-analysis on intergenerational transmission of literacy. Rev Educ Res. 65(1):1–21. doi: 10.3102/00346543065001001.

[ref10] Cao F, Bitan T, Booth JR. 2008. Effective brain connectivity in children with reading difficulties during phonological processing. Brain Lang. 107(2):91–101. doi: 10.1016/j.bandl.2007.12.009. 18226833 PMC2676797

[ref11] Cheng CH, Chan PYS, Hsieh YW, Chen KF. 2016. A meta-analysis of mismatch negativity in children with attention deficit-hyperactivity disorders. Neurosci Lett. 612:132–137. doi: 10.1016/j.neulet.2015.11.033. 26628248

[ref12] Cheour M, Korpilahti P, Martynova O, Lang A-H. 2001. Mismatch negativity and late discriminative negativity in investigating speech perception and learning in children and infants. Audiol Neurotol. 6(1):2–11. doi: 10.1159/000046804. 11173771

[ref14] Cronin V, Carver P. 1998. Phonological sensitivity, rapid naming, and beginning reading. Appl Psycholinguist. 19(3):447–461. doi: 10.1017/S0142716400010262.

[ref15] Dalmaijer ES, Bignardi G, Anwyl-Irvine AL, Smith TA, Siugzdaite R, Uh S, Johnson A, Astle D. 2019. Direct and indirect links between children’s socio-economic status and education: pathways via mental health, attitude, and cognition [preprint]. PsyArXiv. doi: 10.31234/osf.io/yfn56. PMC7614555 37215737

[ref16] Dehaene-Lambertz G, Gliga T. 2004. Common neural basis for phoneme processing in infants and adults. J Cogn Neurosci. 16(8):1375–1387. doi: 10.1162/0898929042304714. 15509385

[ref17] Dolean D, Melby-Lervåg M, Tincas I, Damsa C, Lervåg A. 2019. Achievement gap: socioeconomic status affects reading development beyond language and cognition in children facing poverty. Learn Instr. 63:101218. doi: 10.1016/j.learninstruc.2019.101218.

[ref19] Fischl B . 2012. FreeSurfer. NeuroImage. 62(2):774–781. doi: 10.1016/j.neuroimage.2012.01.021. 22248573 PMC3685476

[ref20] Furnes B, Samuelsson S. 2011. Phonological awareness and rapid automatized naming predicting early development in reading and spelling: results from a cross-linguistic longitudinal study. Learn Individ Differ. 21(1):85–95. doi: 10.1016/j.lindif.2010.10.005. 21359098 PMC3045196

[ref21] Friston KJ, Worsley KJ, Frackowiak RS, Mazziotta JC, Evans AC. 1994. Assessing the significance of focal activations using their spatial extent. Hum Brain Mapp. 1(3):210–220. 24578041 10.1002/hbm.460010306

[ref22] Gallagher A, Frederickson N. 1995. The phonological assessment battery (PhAB): an initial assessment of its theoretical and practical utility. Educ Child Psychol. 12(1):53–67.

[ref23] Garcia-Sierra A, Rivera-Gaxiola M, Percaccio CR, Conboy BT, Romo H, Klarman L, Ortiz S, Kuhl PK. 2011. Bilingual language learning: an ERP study relating early brain responses to speech, language input, and later word production. J Phon. 39(4):546–557. doi: 10.1016/j.wocn.2011.07.002.

[ref24] Gramfort A, Luessi M, Larson E, Engemann DA, Strohmeier D, Brodbeck C, Goj R, Jas M, Brooks T, Parkkonen L, et al. 2013. MEG and EEG data analysis with MNE-python. Front Neurosci. 7. doi: 10.3389/fnins.2013.00267. PMC3872725 24431986

[ref101] Hawkins E., Astle DE., Rastle K. 2015. Semantic advantage for learning new phonological form representations. J Cogn Neurosci. 27(4):775–786. 25269110 10.1162/jocn_a_00730

[ref25] Hill PR, McArthur GM, Bishop DVM. 2004. Phonological categorization of vowels: a mismatch negativity study. NeuroReport. 15(14):2195–2199. 15371732 10.1097/00001756-200410050-00010

[ref26] Hoff-Ginsberg E . 1998. The relation of birth order and socioeconomic status to children’s language experience and language development. Appl Psycholinguist. 19(4):603–629. doi: 10.1017/S0142716400010389.

[ref27] Hulme C, Snowling M, Caravolas M, Carroll J. 2005. Phonological skills are (probably) one cause of success in learning to read: a comment on Castles and Coltheart. Sci Stud Read. 9(4):351–365. doi: 10.1207/s1532799xssr0904_2.

[ref28] Junge C, Cutler A, Hagoort P. 2012. Electrophysiological evidence of early word learning. Neuropsychologia. 50(14):3702–3712. doi: 10.1016/j.neuropsychologia.2012.10.012. 23108241

[ref30] Kim H . 2014. Involvement of the dorsal and ventral attention networks in oddball stimulus processing: a meta-analysis. Hum Brain Mapp. 35(5):2265–2284. doi: 10.1002/hbm.22326. 23900833 PMC6868981

[ref31] Kishiyama MM, Boyce WT, Jimenez AM, Perry LM, Knight RT. 2009. Socioeconomic disparities affect prefrontal function in children. J Cogn Neurosci. 21(6):1106–1115. doi: 10.1162/jocn.2009.21101. 18752394

[ref32] Kolenikov S, Angeles G. 2009. Socioeconomic status measurement with discrete proxy variables: is principal component analysis a reliable answer? Rev Income Wealth. 55(1):128–165. doi: 10.1111/j.1475-4991.2008.00309.x.

[ref33] Korpilahti P, Krause CM, Holopainen I, Lang AH. 2001. Early and late mismatch negativity elicited by words and speech-like stimuli in children. Brain Lang. 76(3):332–339. doi: 10.1006/brln.2000.2426. 11247648

[ref34] Krajewski K, Schneider W. 2009. Exploring the impact of phonological awareness, visual–spatial working memory, and preschool quantity–number competencies on mathematics achievement in elementary school: findings from a 3-year longitudinal study. J Exp Child Psychol. 103(4):516–531. doi: 10.1016/j.jecp.2009.03.009. 19427646

[ref35] Lage-Castellanos A, Martínez-Montes E, Hernández-Cabrera JA, Galán L. 2010. False discovery rate and permutation test: an evaluation in ERP data analysis. Stat Med. 29(1):63–74. 19941298 10.1002/sim.3784

[ref36] Linnavalli T, Putkinen V, Huotilainen M, Tervaniemi M. 2017. Phoneme processing skills are reflected in children’s MMN responses. Neuropsychologia. 101:76–84. doi: 10.1016/j.neuropsychologia.2017.05.013. 28506807

[ref37] Lovio R, Pakarinen S, Huotilainen M, Alku P, Silvennoinen S, Näätänen R, Kujala T. 2009. Auditory discrimination profiles of speech sound changes in 6-year-old children as determined with the multi-feature MMN paradigm. Clin Neurophysiol. 120(5):916–921. doi: 10.1016/j.clinph.2009.03.010. 19386542

[ref38] Lyytinen H, Blomberg AP, Näätänen R. 1992. Event-related potentials and autonomic responses to a change in unattended auditory stimuli. Psychophysiology. 29(5):523–534. doi: 10.1111/j.1469-8986.1992.tb02025.x. 1410181

[ref39] Lung FW, Shu BC, Chiang TL, Lin SJ. 2009. Parental mental health, education, age at childbirth and child development from six to 18 months. Acta Paediatr. 98(5):834–841. 19120038 10.1111/j.1651-2227.2008.01166.x

[ref40] Maurer U, Bucher K, Brem S, Brandeis D. 2003. Development of the automatic mismatch response: from frontal positivity in kindergarten children to the mismatch negativity. Clin Neurophysiol. 114(5):808–817. doi: 10.1016/S1388-2457(03)00032-4. 12738427

[ref41] McCrimmon AW, Smith AD. 2013. Review of the Wechsler abbreviated scale of intelligence, second edition (WASI-II). J Psychoeduc Assess. 31(3):337–341. doi: 10.1177/0734282912467756.

[ref42] McLoyd VC . 1998. Socioeconomic disadvantage and child development. Am Psychol. 53(2):185–204. doi: 10.1037//0003-066x.53.2.185. 9491747

[ref43] Meador KJ, Hammond EJ, Loring DW, Feldman DS, Bowers D, Heilman KM. 1987. Auditory P3 correlates of phonemic and semantic processing. Int J Neurosci. 35(3–4):175–179. doi: 10.3109/00207458708987125. 3654074

[ref44] Näätänen R . 1990. The role of attention in auditory information processing as revealed by event-related potentials and other brain measures of cognitive function. Behav Brain Sci. 13(2):201–233. doi: 10.1017/S0140525X00078407.

[ref45] Naccache L, Dehaene S. 2001. The priming method: imaging unconscious repetition priming reveals an abstract representation of number in the parietal lobes. Cereb Cortex. 11(10):966–974. doi: 10.1093/cercor/11.10.966. 11549619

[ref46] Nation K, Snowling MJ. 2004. Beyond phonological skills: broader language skills contribute to the development of reading. J Res Read. 27(4):342–356. doi: 10.1111/j.1467-9817.2004.00238.x.

[ref47] Nichols T, Hayasaka S. 2003. Controlling the familywise error rate in functional neuroimaging: a comparative review. Stat Methods Med Res. 12(5):419–446. 14599004 10.1191/0962280203sm341ra

[ref48] Noble KG, Houston SM, Kan E, Sowell ER. 2012. Neural correlates of socioeconomic status in the developing human brain. Dev Sci. 15(4):516–527. doi: 10.1111/j.1467-7687.2012.01147.x. 22709401 PMC6554027

[ref49] Noble KG, McCandliss BD, Farah MJ. 2007. Socioeconomic gradients predict individual differences in neurocognitive abilities. Dev Sci. 10(4):464–480. doi: 10.1111/j.1467-7687.2007.00600.x. 17552936

[ref50] Noble KG, Norman MF, Farah MJ. 2005. Neurocognitive correlates of socioeconomic status in kindergarten children. Dev Sci. 8(1):74–87. doi: 10.1111/j.1467-7687.2005.00394.x. 15647068

[ref51] Noble KG, Wolmetz ME, Ochs LG, Farah MJ, McCandliss BD. 2006. Brain–behavior relationships in reading acquisition are modulated by socioeconomic factors. Dev Sci. 9(6):642–654. doi: 10.1111/j.1467-7687.2006.00542.x. 17059461

[ref52] Odgers CL, Adler NE. 2018. Challenges for low-income children in an era of increasing income inequality. Child Dev Perspect. 12(2):128–133.

[ref53] Office for National Statistics . 2018. Regional Ethnic Diversity. Ethnicity Facts and Figures, GOV.UK. https://www.ethnicity-facts-figures.service.gov.uk/uk-population-by-ethnicity/national-and-regional-populations/regional-ethnic-diversity/latest (last accessed 27 July 2020).

[ref54] Oram Cardy JE, Flagg EJ, Roberts W, Roberts TPL. 2005. Delayed mismatch field for speech and non-speech sounds in children with autism. NeuroReport. 16(5):521. 15770164 10.1097/00001756-200504040-00021

[ref55] Orinstein AJ, Stevens MC. 2014. Brain activity in predominantly-inattentive subtype attention-deficit/hyperactivity disorder during an auditory oddball attention task. Psychiatry Res Neuroimaging. 223(2):121–128. doi: 10.1016/j.pscychresns.2014.05.012. PMC4120259 24953999

[ref56] Ostrove JM, Adler NE, Kuppermann M, Washington AE. 2000. Objective and subjective assessments of socioeconomic status and their relationship to self-rated health in an ethnically diverse sample of pregnant women. Health Psychol. 19(6):613. 11129365 10.1037//0278-6133.19.6.613

[ref57] Paulson JF, Keefe HA, Leiferman JA. 2009. Early parental depression and child language development. J Child Psychol Psychiatry. 50(3):254–262. 19175819 10.1111/j.1469-7610.2008.01973.x

[ref58] Polich J . 2007. Updating P300: an integrative theory of P3a and P3b. Clin Neurophysiol. 118(10):2128–2148. doi: 10.1016/j.clinph.2007.04.019. 17573239 PMC2715154

[ref59] Pungello EP, Iruka IU, Dotterer AM, Mills-Koonce R, Reznick JS. 2009. The effects of socioeconomic status, race, and parenting on language development in early childhood. Dev Psychol. 45(2):544–557. doi: 10.1037/a0013917. 19271838

[ref60] Rubin M, Denson N, Kilpatrick S, Matthews KE, Stehlik T, Zyngier D. 2014. “I am working-class”: subjective self-definition as a missing measure of social class and socioeconomic status in higher education research. Educ Res. 43(4):196–200. doi: 10.3102/0013189X14528373.

[ref61] Sams M, Aulanko R, Hämäläinen M, Hari R, Lounasmaa OV, Lu ST, Simola J. 1991. Seeing speech: visual information from lip movements modifies activity in the human auditory cortex. Neurosci Lett. 127(1):141–145. doi: 10.1016/0304-3940(91)90914-F. 1881611

[ref62] Sassenhagen J, Draschkow D. 2019. Cluster-based permutation tests of MEG/EEG data do not establish significance of effect latency or location. Psychophysiology. 56(6):e13335. doi: 10.1111/psyp.13335. 30657176

[ref63] Schoon I, Parsons S, Rush R, Law J. 2010. Childhood language skills and adult literacy: a 29-year follow-up study. Pediatrics. 125(3):e459–e466. doi: 10.1542/peds.2008-2111. 20142287

[ref64] Schröger E . 1997. On the detection of auditory deviations: a pre-attentive activation model. Psychophysiology. 34(3):245–257. doi: 10.1111/j.1469-8986.1997.tb02395.x. 9175439

[ref65] Scott KM, Al-Hamzawi AO, Andrade LH, Borges G, Caldas-de-Almeida JM, Kawakami N. 2014. Associations between subjective social status and DSM–IV mental disorders: results from the world mental health surveys. JAMA Psychiat. 71:1400–1408. 10.1001/jamapsychiatry.2014.1337 PMC5315238 25354080

[ref66] Shafer VL, Morr ML, Datta H, Kurtzberg D, Schwartz RG. 2005. Neurophysiological indexes of speech processing deficits in children with specific language impairment. J Cogn Neurosci. 17(7):1168–1180. doi: 10.1162/0898929054475217. 16138434

[ref67] Singh-Manoux A, Marmot MG, Adler NE. 2005. Does subjective social status predict health and change in health status better than objective status? Psychosom Med. 67(6):855–861. 16314589 10.1097/01.psy.0000188434.52941.a0

[ref68] Sommer W, Matt J. 1990. Awareness of P300-related cognitive processes: a signal detection approach. Psychophysiology. 27(5):575–585. doi: 10.1111/j.1469-8986.1990.tb01980.x. 2274621

[ref69] Stevens C, Lauinger B, Neville H. 2009. Differences in the neural mechanisms of selective attention in children from different socioeconomic backgrounds: an event-related brain potential study. Dev Sci. 12(4):634–646. doi: 10.1111/j.1467-7687.2009.00807.x. 19635089 PMC2718768

[ref70] Stevens C, Paulsen D, Yasen A, Neville H. 2015. Atypical auditory refractory periods in children from lower socio-economic status backgrounds: ERP evidence for a role of selective attention. Int J Psychophysiol. 95(2):156–166. doi: 10.1016/j.ijpsycho.2014.06.017. 25003553

[ref71] Takeda Y, Kimura M. 2014. The auditory N1 amplitude for task-irrelevant probes reflects visual interest. Int J Psychophysiol. 94(1):35–41. doi: 10.1016/j.ijpsycho.2014.07.007. 25058330

[ref72] Taylor MJ, Baldeweg T. 2002. Application of EEG, ERP and intracranial recordings to the investigation of cognitive functions in children. Dev Sci. 5(3):318–334.

[ref73] Torgesen JK, Wagner RK, Rashotte CA. 1994. Longitudinal studies of phonological processing and reading. J Learn Disabil. 27(5):276–286. doi: 10.1177/002221949402700503. 8006506

[ref74] Ursache A, Noble KG. 2016. Neurocognitive development in socioeconomic context: multiple mechanisms and implications for measuring socioeconomic status. Psychophysiology. 53(1):71–82. doi: 10.1111/psyp.12547. 26681619 PMC4685721

[ref75] Ursache A, Noble KG, Blair C. 2015. Socioeconomic status, subjective social status, and perceived stress: associations with stress physiology and executive functioning. Behav Med. 41(3):145–154. doi: 10.1080/08964289.2015.1024604. 26332932 PMC4722863

[ref76] Vihman MM . 1996. Phonological development: the origins of language in the child. Oxford, United Kingdom: Blackwell Publishing.

[ref77] Wagner RK, Torgesen JK, Rashotte CA, Hecht SA, Barker TA, Burgess SR, Donahue J, Garon T. 1997. Changing relations between phonological processing abilities and word-level reading as children develop from beginning to skilled readers: a 5-year longitudinal study. Dev Psychol. 33(3):468–479. doi: 10.1037//0012-1649.33.3.468. 9149925

[ref78] Wagner RK, Torgesen JK. 1987. The nature of phonological processing and its causal role in the acquisition of reading skills. Psychol Bull. 101(2):192–212. doi: 10.1037/0033-2909.101.2.192.

[ref79] Wehner DT, Ahlfors SP, Mody M. 2007. Effects of phonological contrast on auditory word discrimination in children with and without reading disability: a magnetoencephalography (MEG) study. Neuropsychologia. 45(14):3251–3262. doi: 10.1016/j.neuropsychologia.2007.06.018. 17675109 PMC2147041

[ref80] Wheldall K, Pogorzelski S. 2003. Is the phab really fab? The utility of the phonological assessment battery in predicting gains made by older low-progress readers following two terms of intensive literacy instruction. Educ Psychol. 23(5):569–590. doi: 10.1080/0144341032000123804.

[ref81] Whitehurst GJ . 1997. Language processes in context: language learning in children reared in poverty. In Research on communication and language disorders: Contribution to theories of language development (pp. 233–266). Baltimore, MA: Brookes.

[ref82] Woodcock RW, McGrew KS, Mather N. 2001. Woodcock-Johnson III Test. Salisbury, United Kingdom.

[ref100] Younger JW., Lee KW., Demir-Liraa OE., Boot JR. 2019. Brain lateralization of phonological awareness varies by maternal education. Dev Sci. 22(6):e12807. 30735285 10.1111/desc.12807

